# Atypical Amygdala–Neocortex Interaction During Dynamic Facial Expression Processing in Autism Spectrum Disorder

**DOI:** 10.3389/fnhum.2019.00351

**Published:** 2019-10-18

**Authors:** Wataru Sato, Takanori Kochiyama, Shota Uono, Sayaka Yoshimura, Yasutaka Kubota, Reiko Sawada, Morimitsu Sakihama, Motomi Toichi

**Affiliations:** ^1^Kokoro Research Center, Kyoto University, Kyoto, Japan; ^2^Brain Activity Imaging Center, ATR-Promotions, Inc., Kyoto, Japan; ^3^Department of Neurodevelopmental Psychiatry, Habilitation and Rehabilitation, Graduate School of Medicine, Kyoto University, Kyoto, Japan; ^4^Health and Medical Services Center, Shiga University, Hikone, Japan; ^5^Faculty of Human Health Science, Graduate School of Medicine, Kyoto University, Kyoto, Japan; ^6^The Organization for Promoting Developmental Disorder Research, Kyoto, Japan; ^7^Rakuwa-kai Otowa Hospital, Kyoto, Japan

**Keywords:** amygdala, autism spectrum disorder (ASD), dynamic causal modeling (DCM), dynamic facial expressions of emotion, functional magnetic resonance imaging (fMRI)

## Abstract

Atypical reciprocal social interactions involving emotional facial expressions are a core clinical feature of autism spectrum disorder (ASD). Previous functional magnetic resonance imaging (fMRI) studies have demonstrated that some social brain regions, including subcortical (e.g., amygdala) and neocortical regions (e.g., fusiform gyrus, FG) are less activated during the processing of facial expression stimuli in individuals with ASD. However, the functional networking patterns between the subcortical and cortical regions in processing emotional facial expressions remain unclear. We investigated this issue in ASD (*n* = 31) and typically developing (TD; *n* = 31) individuals using fMRI. Participants viewed dynamic facial expressions of anger and happiness and their corresponding mosaic images. Regional brain activity analysis revealed reduced activation of several social brain regions, including the amygdala, in the ASD group compared with the TD group in response to dynamic facial expressions vs. dynamic mosaics (*p* < 0.05, $\eta_{\text{p}}^{2}$ = 0.19). Dynamic causal modeling (DCM) analyses were then used to compare models with forward, backward, and bi-directional effective connectivity between the amygdala and neocortical networks. The results revealed that: (1) the model with effective connectivity from the amygdala to the neocortex best fit the data of both groups; and (2) the same model best accounted for group differences. Coupling parameter (i.e., effective connectivity) analyses showed that the modulatory effects of dynamic facial processing were substantially weaker in the ASD group than in the TD group. These findings suggest that atypical modulation from the amygdala to the neocortex underlies impairment in social interaction involving dynamic facial expressions in individuals with ASD.

## Introduction

Individuals with autism spectrum disorder (ASD) exhibit atypical social interactions (American Psychiatric Association, 2013). One of the most evident features of their social atypicality is deficient communication *via* emotional facial expressions (Hobson, 1993). Previous observational studies have reported that individuals with ASD exhibited attenuated emotional behaviors (e.g., Corona et al., 1998) and reduced and/or inappropriate facial reactions (e.g., Yirmiya et al., 1989) in response to others’ facial expressions in social interactions compared with typically developing (TD) individuals. Experimental studies suggested that individuals with ASD are specifically impaired in the processing of dynamic, compared with static, facial expressions. For example, previous studies reported that ASD groups showed atypical perceptual (e.g., Palumbo et al., 2015; Uono et al., 2014), cognitive (e.g., Kessels et al., 2010; Sato et al., 2013), and motor (e.g., Rozga et al., 2013; Yoshimura et al., 2015) reactions during observations of dynamic facial expressions.

Several functional magnetic resonance imaging (fMRI) studies have investigated the neural mechanisms underlying atypical processing of dynamic facial expressions in individuals with ASD (Pelphrey et al., 2007; Rahko et al., 2012; Sato et al., 2012b). Although the results are not consistent across studies, some studies consistently reported that the observation of dynamic facial expressions evoked less activation in ASD groups than in TD groups of some subcortical brain regions, such as the amygdala, and some neocortical regions, such as the fusiform gyrus (FG) and superior temporal sulcus (STS) region (including the adjacent middle and superior temporal gyri; see Allison et al., 2000), and the inferior frontal gyrus (IFG; Pelphrey et al., 2007; Sato et al., 2012b). Abundant neuroimaging and neuropsychological evidence from TD individuals suggests that these brain regions are involved in the specific processing of social stimuli, such as emotional processing in the amygdala (for a review, see Calder et al., 2001), visual analysis of faces in the FG and STS region (for a review, see Haxby et al., 2000), and motor resonance in the IFG (for a review, see Rizzolatti et al., 2001). These regions have been called the “social brain” regions (Brothers et al., 1990; Adolphs, 2003; Blakemore, 2008) and were proposed to be impaired in individuals with ASD (Baron-Cohen et al., 2000; Emery and Perrett, 2000; Johnson et al., 2005; Bachevalier and Loveland, 2006; Frith, 2007; Pelphrey and Carter, 2008). One previous study further investigated functional coupling patterns in the neocortical network during the processing of dynamic facial expressions (Sato et al., 2012b). That study tested the bi-directional network connecting the primary visual cortex (V1), STS region, and IFG using dynamic causal modeling (DCM; Friston et al., 2003). The results showed that the modulatory effects of dynamic expressions on all connections were weaker in the ASD group than in the TD group. Together, these data suggest that a reduction in the activity of subcortical and neocortical social brain regions and their neocortical network may underlie atypical processing of dynamic facial expressions in individuals with ASD.

However, functional networking patterns between the subcortical and neocortical regions during the processing of dynamic facial expressions in individuals with ASD remain unclear, as these previous studies tested the neocortical network only in individuals with ASD. A recent neuroimaging study systematically investigated this issue in TD individuals (Sato et al., 2017b). That study analyzed fMRI data during the observation of dynamic facial expressions using DCM and compared models of the modulatory effects of dynamic facial expressions from the amygdala to the neocortex, from the neocortex to the amygdala, and bi-directionally. The results supported the model of the modulatory effect from the amygdala to the neocortex. This finding is consistent with anatomical evidence in animals that the amygdala receives visual input *via* subcortical pathways bypassing neocortical visual areas (Day-Brown et al., 2010), and sends widespread projections to neocortical regions, including the visual and motor areas (for a review, see Amaral et al., 1992). Several neuroscientific studies in TD individuals have also suggested that the amygdala conducts rapid emotional processing of facial expressions and modulates activities in the neocortical regions (for a review, see Vuilleumier and Pourtois, 2007). Based on these data, together with the aforementioned behavioral findings reporting impaired rapid processing of dynamic facial expressions in individuals with ASD (e.g., perception: Uono et al., 2014), we hypothesized that the modulatory effect from the amygdala to the neocortex may be weaker during the processing of dynamic facial expressions in individuals with ASD than in TD individuals.

In this fMRI study, we tested this hypothesis in a group of individuals with ASD and TD controls while they viewed dynamic facial expressions and their corresponding mosaic images. We analyzed group differences in regional brain activity in response to dynamic facial expressions vs. dynamic mosaics to determine differences in activity in the social brain regions between the ASD and TD groups. We prepared facial expressions of both negative (anger) and positive (happy) valences, though we did not expect different effects across emotions based on previous findings (Sato et al., 2012b). We then conducted DCM and compared models with the modulatory effects of dynamic facial expressions from the amygdala to the neocortex, from the neocortex to the amygdala, and bi-directionally, to determine which model optimally accounted for group commonalities and differences. We predicted that the model with the modulatory effect from the amygdala to the neocortex would be optimal for both purposes.

## Materials and Methods

### Participants

The study included 31 Japanese adults in the ASD group (nine female, 22 male; mean ± *SD* age, 27.2 ± 8.5 years). This group consisted of 23 individuals with Asperger’s disorder (six female, 17 male) and eight with pervasive developmental disorder not otherwise specified (PDD-NOS; three female, five male). Both diagnoses are included within the ASD category in the Diagnostic and Statistical Manual (DSM)-5 (American Psychiatric Association, 2013). PDD-NOS can include the heterogeneous subtypes of ASD, as defined in the DSM-IV-Text Revision (TR; American Psychiatric Association, 2000); only high-functioning PDD-NOS participants with milder symptoms than those associated with Asperger’s disorder were included in this study. The diagnosis was made by at least two psychiatrists with expertise in developmental disorders using the DSM-IV-TR *via* a strict procedure in which every item of the ASD diagnostic criteria was investigated in interviews with participants and their parents (and professionals who helped them, if any). Only participants who met at least one of the four social impairment items without satisfying any items of the criteria of autistic disorder were included. Each participant’s developmental history was assessed through comprehensive interviews. Neurological and psychiatric problems other than those associated with ASD were ruled out. The participants were not taking medication. The intelligence quotients (IQs) of all participants in the ASD group had been assessed at other facilities and were reported to be within the normal range. Participants who agreed to newly undergo IQ tests (*n* = 28) were assessed using the revised Wechsler Adult Intelligence Scale, third edition (Nihon Bunka Kagakusha, Tokyo, Japan) and were confirmed to be in the normal range (full-scale IQ, mean ± *SD*, 110.0 ± 13.4). The symptom severity of the participants who were willing to undergo a further detailed interview (*n* = 25) was assessed quantitatively using the Childhood Autism Rating Scale (Schopler et al., 1986); the scores (mean ± *SD*, 24.4 ± 3.7) were comparable to those from previous studies that included high-functioning individuals with ASD (Koyama et al., 2007; Sato et al., 2012b; Uono et al., 2014; Yoshimura et al., 2015; *t*-test, *p* > 0.1).

The TD control group was comprised of 31 Japanese adults (nine female, 22 male; mean ± *SD* age, 24.2 ± 1.0 years). TD participants had no neurological or psychiatric problems and were matched with the ASD group for age (*t*-test, *p* > 0.1) and sex (*χ*^2^-test, *p* > 0.1). Some of the TD participants agreed to participate in IQ tests (*n* = 27) using the revised Wechsler Adult Intelligence Scale, third edition (Nihon Bunka Kagakusha, Tokyo, Japan) and were confirmed to be in the normal range (full-scale IQ, mean ± *SD*, 121.8 ± 9.7), which was significantly higher than that of the ASD group (*t* = 3.73, *p* < 0.001).

All participants had normal or corrected-to-normal visual acuity and were right-handed, as assessed by the Edinburgh Handedness Inventory (Oldfield, 1971). After the procedures were fully explained, all participants provided written informed consent for participation. This study was approved by the Ethics Committee of the Primate Research Institute, Kyoto University (H2011–05), and was conducted in accordance with the ethical guidelines of the institution.

### Stimuli

Angry and happy facial expressions of eight Japanese models (four female, four male) were presented as video clips. These stimuli were selected from our video database of facial expressions of emotion, which includes 65 Japanese models. The stimulus model looked straight ahead. All faces in the clips were unfamiliar to the participants.

The dynamic expression stimuli consisted of 38 frames ranging from neutral to emotional expressions. Each frame was presented for 40 ms, and each clip was presented for 1,520 ms. The stimuli subtended a visual angle of approximately 15° vertically and 12° horizontally. The validity of these stimuli was supported by previous behavioral findings. Specifically, the speed of these stimuli was demonstrated to sufficiently represent natural changes in dynamic facial expressions (Sato and Yoshikawa, 2004). The stimuli were appropriately recognized as angry and happy expressions (Sato et al., 2010) and elicited appropriate subjective emotional reactions (Sato and Yoshikawa, 2007b) and spontaneous facial mimicry (Sato and Yoshikawa, 2007a; Sato et al., 2008) in TD individuals, but reduced spontaneous facial mimicry in individuals with ASD (Yoshimura et al., 2015).

The dynamic mosaic image stimuli were made from the same materials. All face images were divided into 50 vertical × 40 horizontal squares, which were randomly reordered using a fixed algorithm. This rearrangement made each image unrecognizable as a face. A set of 38 images, corresponding to the original dynamic facial expression stimuli, were presented as a clip at a speed identical to that of the dynamic expression stimuli.

### Apparatus

Experiments were controlled using the Presentation 16.0 software (Neurobehavioral Systems, Albany, CA, USA). Stimuli were projected using a liquid crystal projector (DLA-HD10K; Japan Victor Company, Yokohama, Japan) onto a mirror that was positioned in a scanner in front of the participants. Responses were made using a response box (Response Pad; Current Designs, Philadelphia, PA, USA).

### Procedure

Each participant completed the experimental scanning session, consisting of 20 epochs of 20 s each separated by 20 rest periods (a blank screen) of 10 s each. Each of the four stimulus conditions was presented in different epochs in a pseudorandomized order and the stimuli within each epoch were presented in a randomized order. Each epoch consisted of eight trials; a total of 160 trials were completed by each participant. Stimulus trials were replaced by target trials in eight trials.

During each stimulus trial, a fixation point (a small gray cross on a white background the same size as the stimulus) was presented in the center of the screen for 980 ms. The stimulus was then presented for 1,520 ms. During each target trial, a red cross (1.2° × 1.2°) was presented instead of the stimulus. Participants were instructed to detect the red cross and indicate that they had seen it by pressing a button with the right forefinger as quickly as possible. These dummy tasks ensured that the participants were attending to the stimuli but did not involve any controlled processing of the stimuli. Performance on the dummy target-detection task was perfect (correct identification rate = 100.0%).

### Image Acquisition

Images were acquired using a 3-T scanning system (MAGNETOM Trio, A Tim System; Siemens, Malvern, PA, USA) with a 12-channel head coil. Lateral foam pads were used to stabilize the head position. The functional images consisted of 40 consecutive slices parallel to the anterior–posterior commissure plane, and covered the whole brain. A T2*-weighted gradient-echo echo-planar imaging sequence was used with the following parameters: repetition time (TR) = 2,500 ms; echo time (TE) = 30 ms; flip angle = 90°; matrix size = 64 × 64; voxel size = 3 × 3 × 4 mm. After the acquisition of the functional images, a T1-weighted high-resolution anatomical image was acquired using a magnetization-prepared rapid-acquisition gradient-echo sequence (TR = 2,250 ms; TE = 3.06 ms; TI = 1,000 ms; flip angle = 9°; field of view = 256 × 256 mm; voxel size = 1 × 1 × 1 mm).

### Image Analysis

Image analyses were accomplished using the statistical parametric mapping package SPM12^1^, implemented in the MATLAB R2017b (MathWorks, Natick, MA, USA).

#### Preprocessing

For preprocessing, functional images were realigned using the first scan as a reference to correct for head motion. The realignment parameters revealed only a small (<3 mm) motion correction and no significant difference between the ASD and TD groups (*p* > 0.1 for *x*, *y*, *z*-translation and *x*, *y*, *z*-rotation). Next, all functional images were corrected for slice timing. The functional images were then coregistered to the anatomical image and all anatomical and functional images were normalized to Montreal Neurological Institute space using the anatomical image-based unified segmentation-spatial normalization approach (Ashburner and Friston, 2005). Finally, the normalized functional images were resampled to a voxel size of 2 × 2 × 2 mm and smoothed with an isotopic Gaussian kernel of 8 mm full width at half maximum.

#### Regional Brain Activity Analysis

To ensure that our paradigm engaged the functional anatomy of dynamic facial expression processing—for subsequent dynamic causal modeling, we performed two sets of activation analyses (Supplementary Figure S1). These included a region of interest (ROI) analysis within predefined ROIs and a mass-univariate, whole-brain analysis using statistical parametric mapping.

For these analyses, we performed a two-stage random effects analysis to identify significantly activated voxels at the population level (Holmes and Friston, 1998). First, a subject-level analysis was performed using a general linear model (GLM) framework (Friston et al., 1995). Boxcar functions encoded the main conditions, and Delta or stick functions modeled the target condition. These functions were convolved with a canonical hemodynamic response function. The realignment parameters were used as covariates to account for motion-related noise. We used a high-pass filter with a cut-off period of 128 s to eliminate the artifactual low-frequency trend. Serial autocorrelation was accounted for using a first-order autoregressive model.

Next, second group-level analyses were counducted. Based on our primary interest in analyzing group differences in functional networking patterns, we selected regions previously reported to be activated as ROIs for use in constructing the functional network during the processing of dynamic facial expressions in TD individuals (Sato et al., 2017b). The ROIs specifically included the amygdala, fifth visual area (V5)/middle temporal area (MT), FG, STS, and IFG in the right hemisphere. Although a previous study reported that V5/MT activity during the observation of dynamic facial expressions did not differ between ASD and TD groups (Pelphrey et al., 2007), we included this region because: (1) data from another study testing the observation of dynamic facial expressions suggested reduced activity in this region in the ASD group (Sato et al., 2012b); (2) several fMRI studies testing different types of dynamic social stimuli reported reduced activity in this region in ASD individuals (Herrington et al., 2007; Brieber et al., 2010; Borowiak et al., 2018); and (3) a previous DCM study indicated that the functional network for processing dynamic facial expressions in TD individuals includes this region (Sato et al., 2017b). The coordinates in the Montreal Neurological Institute space of each ROI were derived from the results of this previous study (Sato et al., 2017b) and were identical to those used in the subsequent DCM analysis (Supplementary Figure S2).

The beta value for the effect of interest for each participant was extracted as the first eigenvariate of all voxels within a sphere of 4-mm radius around the participant-specific activation foci. The beta values for all ROIs were then subjected to a multivariate analysis of covariance (MANCOVA) with group (ASD vs. TD) as a between-subject factor, stimulus type (expression vs. mosaic) and emotion (anger vs. happiness) as within-subject factors, and sex and age as effect-of-no-interest covariates. Wilks’ *λ* criterion was used. Significant effects were further tested using *t*-tests for single ROIs. Statistical significance was determined at a level of *p* < 0.05. To investigate possible confounding factors, including full-scale IQ and ASD subgroups, we preliminarily conducted the same multivariate analyses: (1) using full-scale IQ as a covariate among participants for whom we collected IQ data; or (2) substituting one ASD subgroup (Asperger or PDD-NOS) for the full ASD group. Because these analyses obtained similarly significant results, we omitted these factors in the reported results.

We then conducted exploratory analyses for the whole brain. Based on the results of the above ROI analysis, the effects of stimulus type (expression vs. mosaic) were analyzed using a two-sample *t*-test with group (ASD, TD) as an effect of interest and sex (male, female) and age as effects of no interest. Significantly activated voxels were identified if they reached an extent threshold of *p* < 0.05, corrected for multiple comparisons, with a cluster-forming threshold of *p* < 0.001 (uncorrected).

Brain structures were labeled anatomically and identified according to Brodmann’s areas using the Automated Anatomical Labeling (AAL) atlas (Tzourio-Mazoyer et al., 2002) and Brodmann maps (Brodmann.nii), respectively, with the MRIcron tool^2^.

#### DCM

For DCM analysis, we conducted group-level inference using a parametric empirical Bayesian (PEB) approach with the SPM12/DCM12 software (Friston et al., 2016; Zeidman et al., 2019a,b; see Supplementary Figure S1). PEB-DCM involved specifying a hierarchical model with two levels: individual subject and group. At the individual subject level, DCM parameters including neuronal interaction and a hemodynamic model of neurovascular coupling in each region was estimated from the fMRI time series data using variational Bayes under the Laplace approximation (Friston et al., 2003). At the group level, first-level (connectivity) parameters were entered into the second-level GLM to evaluate group effects and between-subjects parameter variability. We adopted the PEB-DCM approach because it offers several advantages over previously applied methods. Theoretically, PEB-DCM allows us to conduct more accurate and robust group inference by taking into account the posterior expectations (i.e., means) of the parameters and their posterior covariance; thus, parameter estimates at the individual subject level are adaptively weighted according to precision. Practically, this approach provides a direct and efficient method of performing group-level Bayesian model comparisons (BMCs) and Bayesian parameter inference to determine which model and connections best explain group differences. PEB-DCM was performed in the following four steps: (1) re-specification of the GLM to construct factor-specific regressors or DCM inputs and extraction of an fMRI time series from each participant; (2) specification of the neural network model space; (3) model estimation [steps (1–3) were performed at the individual subject level]; and (4) model comparison and parameter inference at the group level.

DCM allows for the modeling of three different types of effects in a neural network: (1) driving input, which represents the influence of exogenous input on neural states; (2) fixed connections, which represent baseline (i.e., applicable to all experimental conditions) connectivity among neural states; and (3) modulation of extrinsic (between-region) connections by experimental manipulation. Based on our research questions, we investigated the modulatory effect of dynamic facial expression. To construct driving and modulatory inputs for our DCM analysis, we remodeled the single-subject analyses. The design matrix contained the following two experimental factor-specific regressors: visual input (i.e., dynamic facial expressions and dynamic mosaic images) was the driving input in the DCM, and the dynamic facial expression condition was the modulatory input. Based on the results of the above regional brain activity analysis, emotion (anger vs. happiness) and target detection were included as effects of no interest. Other nuisance regressors (realignment parameters and constant terms), high-pass filters, and serial autocorrelations were applied using the settings described above for whole-brain statistical parametric mapping.

To investigate the direction of amygdala–neocortex functional interaction, seven brain regions in the right hemisphere were selected: the pulvinar (*x*14, *y*-30, *z*0), amygdala (*x*24, *y*-8, *z*-12), primary visual cortex (V1; *x*18, *y*-86, *z*-6), V5/MT (*x*48, *y*-60, *z*0), FG (*x*44, *y*-66, *z*-10), STS (*x*58, *y*-38, *z*14), and IFG (*x*50, *y*18, *z*26). The center coordinates of each ROI were derived from the results of the previous study (Sato et al., 2017b). ROIs were restricted to the right hemisphere because some ROIs showed significant activity only in the right hemisphere (Sato et al., 2017b). The time series for each participant was extracted as the first eigenvariate of all voxels within a sphere of 4-mm radius around participant-specific activation foci, within the above ROIs. Participant-specific maxima for each region were selected using the following anatomical and functional criteria. The coordinates for the pulvinar were derived from within a sphere of 4-mm radius around the center coordinates used in the previous study. The coordinates for the amygdala were derived from within the intersection of a sphere of 8-mm radius around the center coordinates used in the previous study and the anatomically defined amygdala mask (Amygdala R in AAL atlas). The coordinates for the V1 were derived from the intersection of a sphere of 16-mm radius around the center coordinates used in the previous study and the anatomically defined calcarine sulcus (Calcarine R in the AAL atlas). The coordinates for the V5/MT, FG, STS and IFG were all derived within a sphere of 8-mm radius around the center coordinates used in the previous study. If no participant-specific maxima were identified, the center coordinates used in the previous study were used as the individual coordinates for that participant. Time-series data were adjusted for effects of no interest and nuisance regressors, high-pass filtered, and corrected for serial correlation.

**Figure 1:**
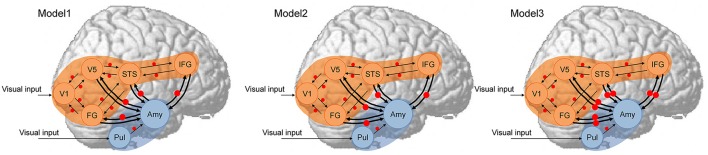
Dynamic causal models. The analyzed brain regions are rendered on spatially normalized brains. Arrows indicate extrinsic connections between brain regions. Red points indicate modulatory effects of dynamic expression. Blue and orange regions indicate subcortical and neocortical subnetworks, respectively, both of which have the same structure across models. Amy, amygdala; FG, fusiform gyrus; IFG, inferior frontal gyrus; STS, superior temporal sulcus; Pul, pulvinar; V1, primary visual cortex; V5, fifth visual area/middle temporal area.

Next, hypothesized models (Figure 1) were constructed for each participant. As a first assumption, the neocortical network, which had a driving input into the V1, and the bi-directional (i.e., forward and backward) extrinsic (between-region) connections of V1–V5/MT, V1–FG, V5/MT–STS, FG–STS, and STS–IFG were all estimated, and the modulatory effect of dynamic facial expression on all extrinsic connections was modeled. This neocortical network was constructed based on the theoretical proposals in the two-pathway model (Oram and Perrett, 1996) and the mirror neuron system model (Hamilton, 2008) for processing dynamic social signals. This neocortical network was validated in the previous study in TD individuals (Sato et al., 2017b), and a similar (partially simplified) model was also validated in ASD individuals (Sato et al., 2012b). As a second assumption, the subcortical network, which had a driving input into the pulvinar, and the forward extrinsic connection of the pulvinar–amygdala were estimated, and the modulatory effect of dynamic presentation on this extrinsic connection was estimated. This subcortical network was constructed based on theoretical (e.g., Vuilleumier, 2005) and empirical (e.g., Morris et al., 1999) evidence for processing emotional facial expressions. Although these studies posited that the superior colliculus sends input to the pulvinar, we did not include the superior colliculus in our model because this region was located adjacent to the pulvinar, making these regions difficult to dissociate using the defined ROI selection method. As a third assumption, we tested the connectivity between the amygdala and the V5/MT, FG, STS, and IFG neocortical regions. We made this assumption because several previous fMRI studies reported a functional interaction between the amygdala and these regions, which was consistent with the results of a previous study in TD individuals (Foley et al., 2012; Sato et al., 2017b). Based on the direction of modulatory effects, we constructed the three models (Figure 1): Model 1 had modulatory connectivity from the amygdala to the neocortex; Model 2 had modulatory connectivity from the neocortex to the amygdala; and Model 3 had bi-directional modulatory connectivity between the amygdala and neocortex.

DCM models were estimated using the FULL + BMR option, which is the default estimation type for DCM12. We estimated only the full-model (in this case, Model 3) parameters for each subject; those of the reduced models (Models 1 and 2) were rapidly computed from the estimated parameters of the full model using a Bayesian model reduction (BMR; Friston et al., 2016).

To examine the direction of amygdala–neocortex connectivity, which best accounted for commonalities and differences across groups, we performed BMC among the three hypothesized models using the second-level PEB-DCM framework (Friston et al., 2016). Based on our research questions, we entered the eight modulatory parameters of amygdala–neocortex interaction from the B matrix of each DCM into the second-level GLM. The second level design matrix consisted of four regressors: the first regressor was a constant term representing commonalities across subjects and second regressor encoded group differences. Two covariates, sex and age, were added to the design matrix as effects of no interest. All regressors except for the first were mean-centered, allowing interpretation of the first regressor as a group mean across subjects. Posterior probability, a BMC evaluation measure, was computed for the three different models with a combination of the two group effects (commonalities and differences) using Bayesian model reduction.

To evaluate the group mean and differences in effective connectivity, we additionally calculated parameter estimates of the averaged model resulting from Bayesian model averaging (BMA). We used the entire model space for averaging, computing weighted averages of each model parameter for which the weighting was provided by the posterior probability for each model (Penny et al., 2010). We thus obtained eight parameter estimates for the modulatory connection of amygdala–neocortex interaction, which were evaluated using the posterior probability of models with and without each parameter.

## Results

### Regional Brain Activity

**Figure 2:**
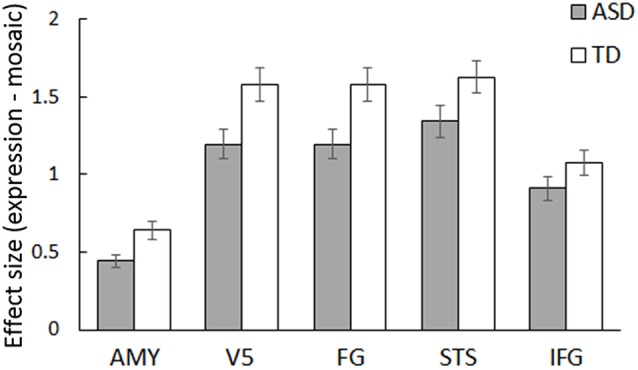
Mean (± SE) beta value for the main effect of stimulus type (dynamic expression vs. dynamic mosaic) in autism spectrum disorder (ASD) and typically developing (TD) groups in the social brain regions, including the amygdala (AMY), fifth visual area/middle temporal area (V5), fusiform gyrus (FG), superior temporal sulcus region (STS), and inferior frontal gyrus (IFG).

ROI analyses were conducted for predefined social brain regions, including the amygdala, V5, FG, STS region, and IFG, using a MANCOVA with group, stimulus type, and emotion as factors and sex and age as covariates. The results revealed a significant interaction between group and stimulus type (*F*_(5,54)_ = 2.58, *p* < 0.05, $\eta_{\text{p}}^{2}$ = 0.19). Besides, only the main effect of stimulus type was significant (*F*_(5,54)_ = 4.28, *p* < 0.005, $\eta_{\text{p}}^{2}$ = 0.28); other main effects and interactions were not significant (*p* > 0.1, $\eta_{\text{p}}^{2}$ < 0.13). The interaction between group and stimulus type indicates that activity in response to dynamic facial expressions vs. dynamic mosaics in these regions differed between the ASD and TD groups; the activity profile showed reduced activity in the ASD group (Figure 2). Follow-up univariate *t*-tests for the difference between dynamic facial expressions vs. dynamic mosaics confirmed significantly reduced activity in the ASD group compared with that in the TD group in the amygdala, V5/MT, and FG (*t*_(60)_ > 2.08; *p* < 0.05), although group differences were marginally significant in the STS region (*t*_(60)_ = 1.54; *p* < 0.1) and not significant in the IFG (*t*_(60)_ = 0.61; *p* > 0.1; Supplementary Figure S2). Whole-brain analyses detected no other significant activation associated with group differences.

### DCM

**Figure 3:**
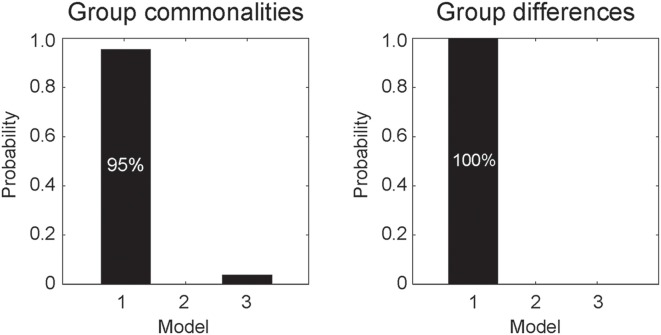
Posterior probabilities of the models, accounting for group commonalities (left) and differences (right) between the ASD and TD groups determined by dynamic causal modeling (DCM) analysis. Model 1, which incorporates the modulatory effect of dynamic expression from the amygdala to the neocortex, best accounted for both commonalities and differences.

DCM analyses were conducted to compare the three network models having different modulatory effects of dynamic expression between the amygdala and neocortical regions (Figure 1). The posterior probability of PEB-DCM analysis indicated that Model 1 with the modulatory effect from the amygdala to the neocortex best accounted for both commonalities and differences among the ASD and TD groups (Figure 3).

**Figure 4:**
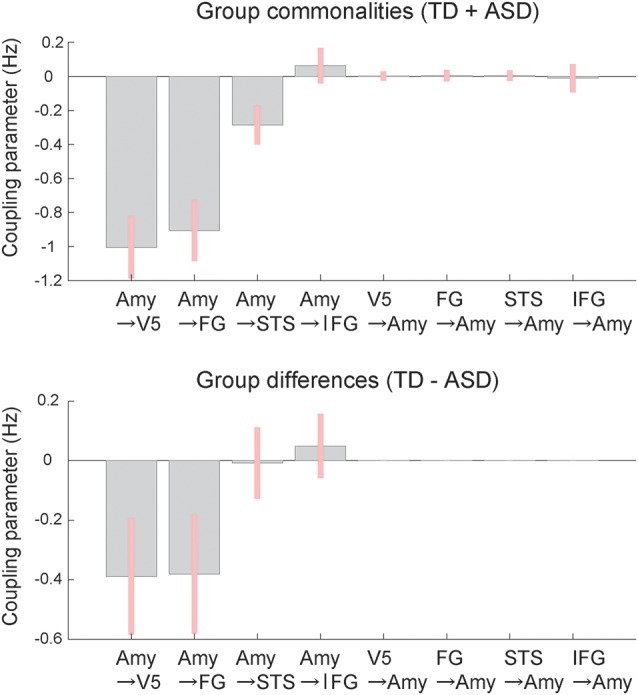
Mean coupling parameters of the modulatory effect of dynamic expression for group commonalities (upper) and differences (lower) between the ASD and TD groups determined by DCM analysis. Pink bars indicate 90% Bayesian credible intervals. Amy, amygdala; FG, fusiform gyrus; IFG, inferior frontal gyrus; STS, superior temporal sulcus; V5, fifth visual area/middle temporal area.

BMA analysis was conducted to inspect profiles of the modulatory effect of dynamic expression. The resultant posterior means of modulatory effect parameters (Figure 4) showed that, with respect to commonalities across groups, the modulatory effects of dynamic facial expression were evident from the amygdala to the neocortex compared with connectivity from the neocortex to the amygdala. Modulatory effects from the amygdala were negative for connectivity to the V5, FG, and STS region and positive for connectivity to the IFG. For all connections from the amygdala to the neocortex, the modulatory effects of dynamic facial expression were weaker (i.e., near zero) in the ASD group than in the TD group.

## Discussion

Our regional brain activity analyses revealed that activity in the social brain regions was collectively lower in the ASD group than in the TD group in response to dynamic facial expressions vs. dynamic mosaic images. The reduced activity of social brain regions in response to dynamic facial expressions in individuals with ASD was largely consistent with the findings of previous studies (Pelphrey et al., 2007; Sato et al., 2012b). However, our results did not show clear group differences in activity in the STS region or IFG, which was not consistent with previous findings, perhaps due to methodological differences. Specifically, the participants in this study were all high-functioning, not taking medication, and without severe symptoms; hence, their ASD traits may have been weaker than those in typical ASD individuals. Together with previous findings, our results suggest that social brain region activity during the processing of dynamic facial expressions is reduced in individuals with ASD.

More important, our DCM analysis provided interesting information regarding the functional networking patterns between the amygdala and neocortical regions during the processing of dynamic facial expressions in individuals with ASD. First, the model with the modulatory effect of dynamic expression from the amygdala to the neocortex best accounted for group commonalities. The results are consistent with previous findings in TD individuals (Sato et al., 2017b). Second, the same model best accounted for differences between the ASD and TD groups. Coupling parameter profiles revealed that the ASD group had weaker modulatory effects than the TD group. Differences in the functional networking patterns observed in ASD individuals were consistent with the previous finding that the modulatory effect of dynamic expression in the neocortical network was weaker in the ASD group than in the TD group (Sato et al., 2012b). However, the previous study did not investigate the functional network between the amygdala and neocortical regions. To the best of our knowledge, these results represent the first evidence that modulatory effects from the amygdala to the neocortex are reduced in individuals with ASD during the processing of dynamic facial expressions.

The coupling parameter profiles showed that the modulatory effects of dynamic facial expressions relative to dynamic mosaics were negative from the amygdala to the V5, FG, and STS region and positive from the amygdala to the IFG in both the ASD and TD groups. These patterns are not necessarily consistent with those reported in the previous study of TD individuals, which showed positive modulatory effects of dynamic facial expressions from the amygdala to all neocortical regions (Sato et al., 2017b). We speculate that this discrepancy may be due to methodological differences between studies, such as the use of stimulus facial expressions of same-race models rather than other-race models, or the use of dynamic mosaic images rather than static facial expressions as the control condition. Similarly, numerous previous studies have investigated functional coupling between the amygdala and posterior neocortical regions during the facial and/or emotional tasks and reported mixed findings, including positive (e.g., Foley et al., 2012; Diano et al., 2017; Jansma et al., 2014) and negative (e.g., Das et al., 2005; Williams et al., 2006; Pantazatos et al., 2012) modulation. These data suggest that the modulatory influence from the amygdala to the posterior neocortical regions may change depending on experimental conditions.

The findings of the present study, together with other neuroscientific evidence, may provide a mechanistic understanding of behavioral problems involving facial expression processing in individuals with ASD. Previous neuroimaging and electrophysiological findings in TD individuals have suggested that the amygdala rapidly conducts emotional processing of facial expressions, because the amygdala is activated by visual input *via* subcortical pathways prior to conscious awareness of the expressions (Morris et al., 1999; Pasley et al., 2004; Williams et al., 2006) specifically at about 100 ms (Bayle et al., 2009; Hung et al., 2010; Sato et al., 2011). A previous DCM analysis of electrophysiological data in TD individuals indicated that the modulation of dynamic facial expressions from the amygdala to the neocortex occurs rapidly at around 200 ms (Sato et al., 2017b). Together with these data, our observation of the reduced modulatory effect from the amygdala to the neocortex in ASD individuals may indicate impaired rapid emotional modulation in widespread neocortical processing for facial expressions, which may partly account for previous behavioral findings that individuals with ASD showed atypical perceptual, cognitive, and motor processing for emotional facial expressions (e.g., Yoshimura et al., 2015).

Our findings may have theoretical implications for the neural mechanisms of social atypicalities in ASD. Several researchers have proposed the theory that individuals with ASD have atypical activity and connectivity in the social brain regions (e.g., Baron-Cohen et al., 2000). However, empirical support for this remains controversial (for reviews, see Müler and Fishman, 2018; Sato and Uono, 2019). Several neuroimaging studies have provided positive evidence for reduced activity in the social brain regions during social stimulus processing. For example, Ciaramidaro et al. (2018) investigated brain activity during implicit and explicit processing of photographs of emotional facial expressions in ASD and TD groups. Implicit, but not explicit, processing of emotional facial expressions were associated with weaker activity in several social brain regions, including the FG, STS region, and amygdala in the ASD group than in the TD group. Sato et al. (2017a) reported reduced activation of the amygdala in response to subliminally presented averted eye gaze in the ASD group. However, other studies reported null or contradictory patterns of social brain region activity. For example, Tottenham et al. (2014) found stronger amygdala activity during the observation of facial expression photographs in the ASD group than in the TD group. Therefore, it may be difficult to draw conclusions about activity in the social brain regions in individuals with ASD. In contrast, a relatively small number of studies have accumulated a positive evidence for reduced functional coupling of the social brain regions in ASD. For example, Ciaramidaro et al. (2015) measured brain activity in response to social films in ASD and TD groups and found reduced functional connectivity between the FG and STS region in the ASD group. Borowiak et al. (2018) reported several reduced functional connections, including between the V5/MT and STS region, during the observation of visual speech in the ASD group. Together with these data, our data suggest that further investigation of the atypical social brain network theory of ASD may be worthwhile, specifically regarding atypical networking patterns in individuals with ASD.

Our finding of atypical amygdala modulation of the widespread neocortical network also has a practical implication. These data suggest the possibility that improvement in amygdala activity may have positive effects on various types of perceptual, cognitive, or motor processing for facial expressions. One previous study has reported that electrical stimulation of the amygdala in individuals with ASD modified their autistic symptoms and face-to-face interactions (Sturm et al., 2013). The effect of oxytocin on ASD symptoms may also be relevant. Previous behavioral studies in individuals with ASD have shown that intranasal administration of oxytocin improved their facial expression processing, including rapid perceptual processing (Xu et al., 2015; Domes et al., 2016). Because neuroimaging studies in TD individuals showed that administration of oxytocin modulates amygdala activity during the processing of emotional facial expressions (Domes et al., 2007; Kanat et al., 2015), we speculate that the modulatory effect from the amygdala to the neocortex may account for the behavioral effect of oxytocin in individuals with ASD. Future research might further examine the effect of electric or pharmacological intervention on amygdala activity to influence various types of social processing *via* modulation of neocortical activity in individuals with ASD.

Several limitations of the present study should be acknowledged. First, IQ was not assessed in all participants. Although we acquired IQ data from most members of the ASD and TD groups and our preliminary analyses suggested that IQ was not related to the patterns of regional brain activity, this finding is not conclusive. This issue could be critical, as previous behavioral studies have suggested that IQ differences between ASD and TD groups may affect differences in the recognition of emotional facial expressions (Harms et al., 2010). Second, the ASD group included heterogeneous subgroups (i.e., Asperger’s disorder and PDD-NOD). Although our preliminary analyses suggested similar patterns of regional brain activity across these subgroups, our sample was too small to investigate this issue. Third, dynamic mosaic images were presented as control stimuli; it remains unclear which types of information processing might reveal group differences in activity and connectivity of the social brain regions. Although dynamic mosaic stimuli could act as control stimuli for dynamic facial expressions in terms of low-level visual properties, such as brightness and motion, and have been used in several previous neuroimaging studies (e.g., De Winter et al., 2015), different types of control stimuli are required to identify specific cognitive or emotional factors associated with group differences in social brain network functioning. Finally, only angry and happy facial expressions were tested. To demonstrate the generalizability of the present findings, investigations of facial expressions of various types of emotions (e.g., Tottenham et al., 2014) are needed. Furthermore, because the amygdala is active during the processing of emotionally neutral faces (Ishai et al., 2005; Sato et al., 2012a), we speculate that the atypical amygdala–neocortex modulation in individuals with ASD may be related to their atypical processing of non-emotional facial actions (e.g., Williams et al., 2004). Additional studies investigating these unsettled issues are required to deepen our understanding of the functioning of the social brain network in individuals with ASD.

In conclusion, our regional brain activity analysis revealed a reduced activity of several social brain regions in response to dynamic facial expressions vs. dynamic mosaic images in the ASD group relative to the TD group. Our DCM analyses revealed that the model with effective connectivity from the amygdala to the neocortex best accounted for commonalities and differences between groups. Modulatory effects were weaker in the ASD group than in the TD group. These results suggest that atypical modulation from the amygdala to the neocortex underlie impairment in social interaction involving dynamic facial expressions in individuals with ASD.

## Data Availability Statement

The datasets generated for this study are available on request to the corresponding author.

## Ethics Statement

This study was approved by the Ethics Committee of the Primate Research Institute, Kyoto University, and was conducted in accordance with the ethical guidelines of the institution. After the experimental procedures had been fully explained, written informed consent was obtained from all participants.

## Author Contributions

WS, TK, SU and MT designed the research. WS, TK, SY and MT analyzed the data. All authors obtained the data, wrote the manuscript, read and approved the final manuscript.

## Conflict of Interest

The authors declare that the research was conducted in the absence of any commercial or financial relationships that could be construed as a potential conflict of interest.

## Footnotes

^1^https://www.fil.ion.ucl.ac.uk/spm

^2^http://www.mccauslandcenter.sc.edu/mricro/mricron/

## Supplementary Material

FIGURE S1 Flowchart for data analysis. fMRI data were acquired from the subjects **(A)**. A general linear model (GLM) with four conditions of interest [i.e., dynamic facial expressions of anger (DyAn) and happiness (DyHa) and their corresponding mosaic images (MoAn, MoHa)] was estimated for each individual subject **(B)**. A multivariate analysis of covariance (MANCOVA) was conducted on the beta estimates of five regions of interest and four conditions **(C)**. In the effective connectivity analysis, time series data **(F)** were extracted from seven volumes of interest (VOIs; **E**) using the rearranged GLM **(D)**. Dynamic causal modeling (DCM) was conducted on three network models encoding the sub–neocortex interaction for individual subjects **(G)**. The estimated DCMs for all subjects were entered into the second-level parametric empirical Bayesian (PEB)-DCM engine **(H)**. Bayesian model comparisons **(J)** and parameter inferences **(K)** were accomplished to evaluate group effects (commonalities and differences across groups) based on the second-level design matrix **(I)**.

FIGURE S2 Regions of interest (ROIs) for regional brain activity and dynamic causal modeling analyses for the amygdala (AMY), fifth visual area/middle temporal (V5), fusiform gyrus (FG), superior temporal sulcus region (STS), and inferior frontal gyrus (IFG) rendered on the spatially normalized brain of a representative participant. The coordinates of each ROI are in the Montreal Neurological Institute space and were derived from the results of Sato et al. (2017b). Activation indicating an interaction between group (TD vs. ASD) and stimulus type (expression vs. mosaic), based on follow-up univariate *t*-tests of a multivariate analysis of covariance, is overlaid in the red–yellow color scale (see the “Results” section).
