# Supplementary figures and images for: Atypical Amygdala–Neocortex Interaction During Dynamic Facial Expression Processing in Autism Spectrum Disorder
Source: Front Hum Neurosci. 2019 Oct 18;13:351. doi: 10.3389/fnhum.2019.00351 (PMC6813184; doi:10.3389/fnhum.2019.00351)

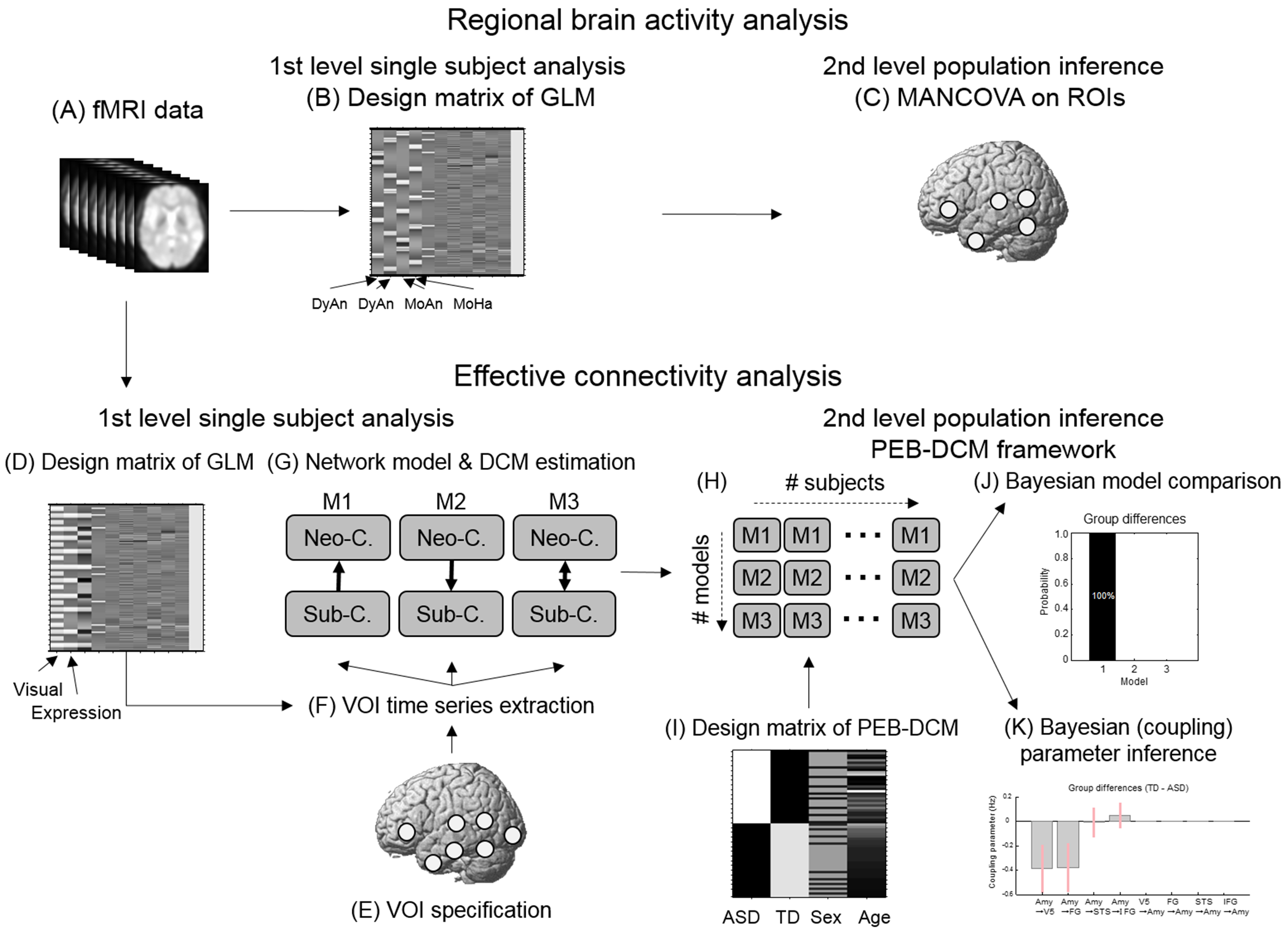

Supplement: FIGURE S1 — Flowchart for data analysis. fMRI data were acquired from the subjects (A). A general linear model (GLM) with four conditions of interest [i.e., dynamic facial expressions of anger (DyAn) and happiness (DyHa) and their corresponding mosaic images (MoAn, MoHa)] was estimated for each individual subject (B). A multivariate analysis of covariance (MANCOVA) was conducted on the beta estimates of five regions of interest and four conditions (C). In the effective connectivity analysis, time series data (F) were extracted from seven volumes of interest (VOIs; E) using the rearranged GLM (D). Dynamic causal modeling (DCM) was conducted on three network models encoding the sub–neocortex interaction for individual subjects (G). The estimated DCMs for all subjects were entered into the second-level parametric empirical Bayesian (PEB)-DCM engine (H). Bayesian model comparisons (J) and parameter inferences (K) were accomplished to evaluate group effects (commonalities and differences across groups) based on the second-level design matrix (I). [file Image_1.TIF]

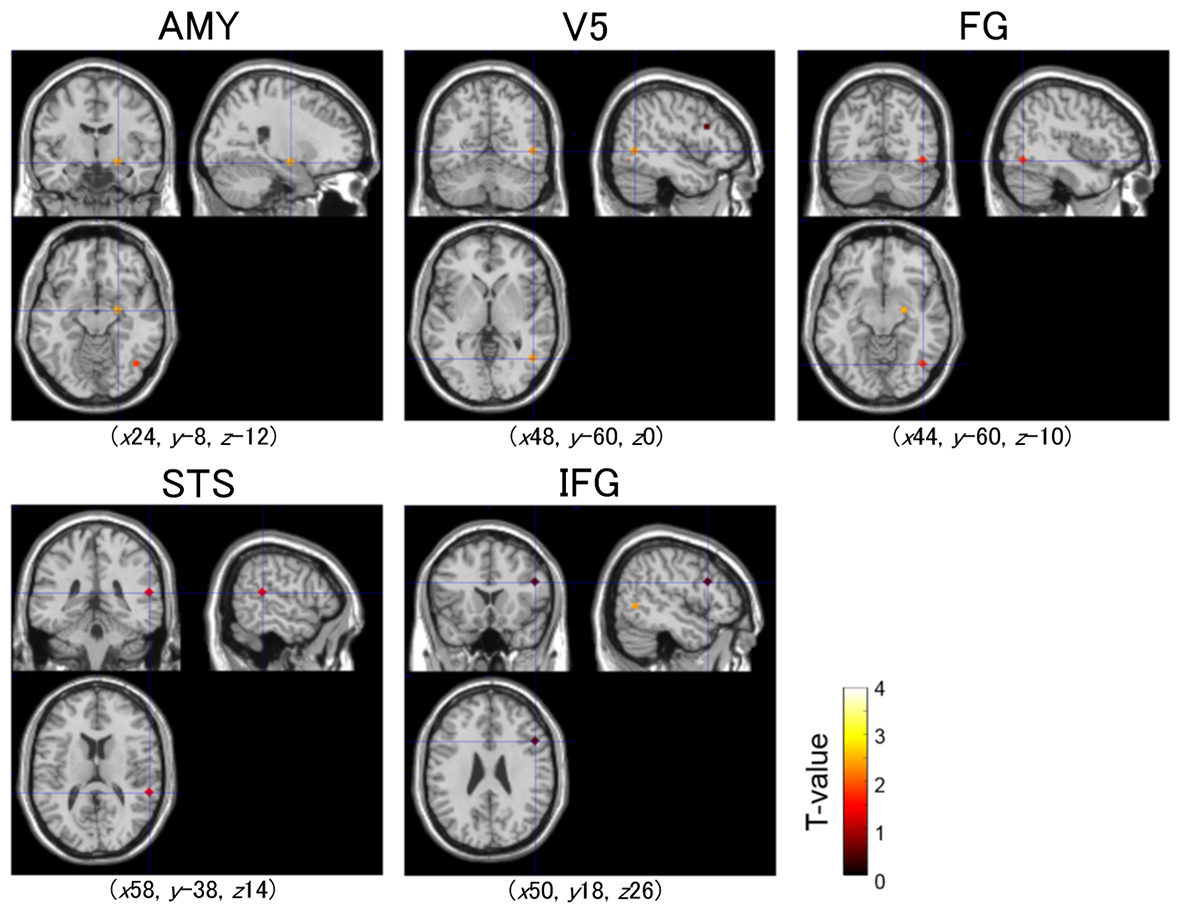

Supplement: FIGURE S2 — Regions of interest (ROIs) for regional brain activity and dynamic causal modeling analyses for the amygdala (AMY), fifth visual area/middle temporal (V5), fusiform gyrus (FG), superior temporal sulcus region (STS), and inferior frontal gyrus (IFG) rendered on the spatially normalized brain of a representative participant. The coordinates of each ROI are in the Montreal Neurological Institute space and were derived from the results of Sato et al. (2017b). Activation indicating an interaction between group (TD vs. ASD) and stimulus type (expression vs. mosaic), based on follow-up univariate t-tests of a multivariate analysis of covariance, is overlaid in the red–yellow color scale (see the “Results” section). [file Image_2.TIF]
